# Tools to implement measurement-based care (MBC) in the treatment of opioid use disorder (OUD): toward a consensus

**DOI:** 10.1186/s13722-024-00446-w

**Published:** 2024-02-28

**Authors:** A. John Rush, Robert E. Gore-Langton, Gavin Bart, Katharine A. Bradley, Cynthia I. Campbell, James McKay, David W. Oslin, Andrew J. Saxon, T. John Winhusen, Li-Tzy Wu, Landhing M. Moran, Betty Tai

**Affiliations:** 1 Duke-NUS Medical School, The National University of Singapore, Duke University School of Medicine, Singapore, Singapore; 2 The Emmes Company, Rockville, MD USA; 3 School of Medicine & Division of Medicine at Hennepin Healthcare, University of Minnesota, Minneapolis, MN USA; 4 Kaiser Permanente Washington Health Research Institute, Seattle, WA USA; 5 Kaiser Permanente Northern California Division of Research, Oakland, CA USA; 6 Penn Center on the Continuum of Care in the Addictions, Philadelphia VA Center of Excellence in Substance Addiction Treatment and Education, University of Pennsylvania, Philadelphia, PA USA; 7 University of Psychiatry, VISN 4 Mental Illness, Research, Education and Clinical Center Crescenz VA Medical Center, Stephen A. Cohen Military Family Clinic at the Perelman School of Medicine, Philadelphia, PA USA; 8 University of Washington and Center of Excellence in Substance Addiction Treatment and Education at the VA Puget Sound Health Care System, Seattle, WA USA; 9 Addiction Sciences, University of Cincinnati College of Medicine, Cincinnati, OH USA; 10 Duke University School of Medicine, Durham, NC USA; 11 Center for Clinical Trials Network, National Institute on Drug Abuse, Bethesda, MD USA; 12 Center for Clinical Trials Network, National Institute on Drug Abuse, National Institutes of Health, 11601 Landsdown Street (3WF), Bethesda, MD 20892 USA

**Keywords:** Measurement-based care, Opioid use disorder, Addiction, Drug, Epidemic, Overdose

## Abstract

**Background:**

The prevalence and associated overdose death rates from opioid use disorder (OUD) have dramatically increased in the last decade. Despite more available treatments than 20 years ago, treatment access and high discontinuation rates are challenges, as are personalized medication dosing and making timely treatment changes when treatments fail. In other fields such as depression, brief measures to address these tasks combined with an action plan—so-called measurement-based care (MBC)—have been associated with better outcomes. This workgroup aimed to determine whether brief measures can be identified for using MBC for optimizing dosing or informing treatment decisions in OUD.

**Methods:**

The National Institute on Drug Abuse Center for the Clinical Trials Network (NIDA CCTN) in 2022 convened a small workgroup to develop consensus about clinically usable measures to improve the quality of treatment delivery with MBC methods for OUD. Two clinical tasks were addressed: (1) to identify the optimal dose of medications for OUD for each patient and (2) to estimate the effectiveness of a treatment for a particular patient once implemented, in a more granular fashion than the binary categories of early or sustained remission or no remission found in The Diagnostic and Statistical Manual of Mental Disorders, fifth edition (DSM-5).

**Discussion:**

Five parameters were recommended to personalize medication dose adjustment: withdrawal symptoms, opioid use, magnitude (severity and duration) of the subjective effects when opioids are used, craving, and side effects. A brief rating of each OUD-specific parameter to adjust dosing and a global assessment or verbal question for side-effects was viewed as sufficient. Whether these ratings produce better outcomes (e.g., treatment engagement and retention) in practice deserves study.

There was consensus that core signs and symptoms of OUD based on some of the 5 DSM-5 domains (e.g., craving, withdrawal) should be the basis for assessing treatment outcome. No existing brief measure was found to meet all the consensus recommendations. Next steps would be to select, adapt or develop de novo items/brief scales to inform clinical decision-making about dose and treatment effectiveness. Psychometric testing, assessment of acceptability and whether the use of such scales produces better symptom control, quality of life (QoL), daily function or better prognosis as compared to treatment as usual deserves investigation.

## Background

### The problem

Treatment delivery for opioid use disorder (OUD) remains challenging for many reasons including low access to treatment and high discontinuation rates [1–3] and the failure to rapidly optimize medication dosing or to make other timely medication changes when outcomes are poor, sometimes referred to as therapeutic or clinical inertia [4]. This workgroup was convened specifically to identify or determine a pathway to develop brief measures for optimizing dosing in measurement-based care (MBC) and to determine treatment effectiveness in patients with OUD.

The term “optimizing dosing” is used herein to mean identifying the medication dose for each patient that provides tolerable side effects with maximal therapeutic effect; the optimal dose varies amongst individual patients. The term “treatment effectiveness” is used consistently throughout this paper to mean there is likelihood that an FDA-approved treatment medication for OUD when administered in clinical practice will provide a measurable benefit to patients with OUD. Treatment effectiveness may have different targets depending on the treatment phase, and thus the phase and target would need to be specified when selecting or developing a measure. For dosage optimization, the target would be symptom control. For treatment effectiveness, targets might be retention, symptom control, functional restoration, relapse (including return to use and misuse) amelioration or some combination of these for an overall assessment. Choice of target(s) must be considered when selecting or developing measures.

The principles of measurement-based care (MBC) were first introduced and extended in the field of depression [5–9] to ensure that each treatment was adequately delivered so that when a treatment failed to produce remission, it was the treatment rather than its delivery that failed. For depression, it entailed a measure of depressive symptoms, a measure of side effects, and an action plan based on these measurements obtained at various times during the trial of the antidepressant [10].

Since then, a plethora of studies in depression and other mental health conditions have supported the value of measuring outcomes to inform treatment decisions for dose adjustment and treatment change [11–14]. While there remain substantial challenges in implementing MBC in daily practice [15], MBC may hold promise for optimizing treatment delivery in patients with OUD—especially for medications [16], thereby enhancing patient retention in treatment and reduction of symptoms and signs of OUD.

To implement MBC in the management of patients with OUD, metrics to optimize dose adjustment (typically an assessment of side effects and the signs and symptoms of the disorder) are needed. The metrics could be a global rating (e.g., a single number) or more granular scales to achieve greater precision. Once the treatment dose has been adjusted (personalized), MBC would require that disorder severity be assessed following a sufficient treatment period to evaluate whether a modification in the type of treatment might be indicated or hopefully that the desired goal has been achieved [7, 9]. In addition, repeated measurements over time, especially in a chronic disease management context, can provide valuable information to predict impending relapses or identify the therapeutic effects of addressing perpetuating factors that affect the outcome of the condition.

Whether MBC efforts like what is used in other chronic diseases would improve outcomes in OUD or for patients with other substance use disorders (SUD) including lower rates of treatment discontinuation or better control of the signs and symptoms has not been well studied. An effectiveness-implementation trial, the measurement-based care to opioid treatment programs project (MBC2OTP), has been launched to inform this important question in opioid treatment programs [17]. The Addiction Medicine Practice-Based Research Network (AMNet) employed a multi-step consensus-based approach for selecting 12 standardized assessment tools and 3 quality of care performance measures for OUD and SUD to facilitate MBC and quality improvement in non-opioid treatment programs [18]. In general, that paper’s objective to support a quality registry including common data elements (CDE) is distinct from those in our paper, as described below.

The workgroup discussions (see Discussion section below) identified two unanswered questions pertinent to the development and assessment of whether MBC methods would improve outcomes in patients with OUD. First, what metric is needed to personalize medication dose adjustment for buprenorphine and other medications to treat OUD? Secondly, what metric is best to judge the overall success of the treatment once implemented for a sufficient period to have its intended effect? The focus was on measures that would be practical in both primary care and specialty addiction treatment settings. The medication most relevant to dosing optimization in primary care in the US is buprenorphine since methadone is administered through opioid treatment programs. However, the consensus decisions reached in this workgroup could apply to all medications for treatment of OUD as may be applicable in other treatment models.

OUD research has largely relied on urine drug testing and sometimes on Timeline Follow-back (TLFB) [19, 20], as outcomes. Urine drug testing, while objective, is a blunt instrument since it is qualitative. It is nearly impossible to distinguish multiple episodes of high-level drug use from a single episode. The likelihood of a positive or negative result is also highly dependent on the timing of the testing in relationship to recent substance use. Further, increasingly primary care visits are virtual, and urine drug screening may add costs to treatment that patients cannot afford. TLFB could conceivably be used in MBC; for example, feasibility for assessing opioid use by an adaptation of TLFB with 4-monthly visits over 16 months of participation has been recently reported [21].

There is not yet wide agreement on one or a few standard simple clinical measures that clinicians and patients could use to either personalize dose adjustments or inform treatment decision-making in the management of each patient. This stands in contrast to the use of scaled measures for alcohol use [22, 23], and many other mental health, neuropsychiatric and general medical conditions, where treatment outcomes are scaled [24]. In OUD treatment trials, domains that are typically measured include: retention in treatment, illicit opioid use as determined by urine toxicology, opioid craving, and global ratings by patient and staff [1]. This scaling of outcomes allows clinicians and patients to gauge the degree of treatment success and thus decide on the next patient-care steps (e.g., change dose, drop or add a treatment, wait longer before making treatment changes, etc.).

The Diagnostic and Statistical Manual of Mental Disorders, fifth edition (DSM-5) indicates that OUD severity can be gauged by summing the number of criterion symptoms used to diagnose OUD that are present over some specified period [25, 26]. Mild severity is ascribed when 2–3, moderate when 4–5, and severe when 6 or more of the 11 criterion symptoms of OUD are present [25, 26].

While documented diagnoses are basically binary measures (i.e., a code is documented or not), the International Classification of Diseases [27] provides some degree of scaling through different codes depending on disorder severity. While these categorical coded outcomes can estimate the overall severity of the condition at the time of diagnosis, they are not granular enough to inform treatment decision making.

The DSM-5 Text Revision (DSM-5-TR) [26] [page 608] indicates that “changing severity across time in an individual is also reflected by reductions in the frequency (e.g., days of use per month) and/or dose (e.g., injections or number of pills) of an opioid, as assessed by the individual self-report, report of knowledgeable others, clinicians’ evaluations, and biological testing.” The period over which either the criterion symptoms should be assessed, or the above metrics of changing severity should be applied, is not specified clearly.

### Previous efforts

In recent years, there have been several efforts to develop and implement scaled outcomes for SUD and OUD, but none have yet gained widespread clinical use [28]. A general substance use symptom checklist has been used across 30 primary care practices and 6 mental health clinics as part of routine care in Kaiser Permanente (KP) Washington since 2015 and, like the alcohol symptom checklist similarly implemented, has demonstrated psychometric validity in patients who screen positive for daily cannabis use or any other drug use [23, 29–33]. Of note, in addition to having psychometric validity, the alcohol and substance use symptoms checklists, as well as questions about alcohol consumption, documented in patients’ electronic health records, have demonstrated reliability [29, 34, 35]. In KP Washington this was implemented as part of the Sustained Patient-centered Alcohol-related Care (SPARC) trial, which used electronic health record (EHR) tools, performance feedback, and locally designed standard workflow and 6 months of quality improvement meetings led by a practice facilitator, to increase Alcohol Use Disorder (AUD) diagnosis and treatment initiation and resulted in sustained screening and assessment with checklists over 4 years later [36, 37]. The count of symptoms is used as a severity measure based on rigorous research on AUD suggesting the count of symptoms is an effective severity measure [38, 39]. However, more recent studies based on DSM-5 severity criteria for AUD indicate there are differences in latent severity associated with AUD symptoms resulting in substantial variability across criteria combinations [40–42] and in subsequent risks [43].

Another relatively straightforward approach has proposed rating each of the DSM-5’s 11 symptoms of OUD and grouping the 11 signs and symptoms into 5 domains: substance use; physiological symptoms; cognitive and behavioral control symptoms; health risks and harms symptoms; and negative social consequences [16]. The authors suggest, as does DSM-5, to score each of the 11 symptoms as present or absent as opposed to rating each symptom on a scale such as a 0–3 rating which would create a long scale (range 0–33) that might be more precise but at the cost of clinical time. The proposal to create 5 domains offers an opportunity to shorten the list from the 11 in the DSM-5.

The Patient-Reported Outcomes Measurement Information System (PROMIS) provides item banks for scaled Severity of Substance Use and Appeal of Substance Use proposed to be suitable for treatment outcome assessment, albeit not specifically for MBC, as well as for observational and epidemiological research [44]. Although the PROMIS Severity of Substance Use bank is relatively long, a Short Form V.1 (form 7a for past 3 months or past 30 days) is available with one binary question on substance use and 7 scaled questions that address 4 of the 11 DSM-5 criteria [45]. This 7-item PROMIS measure [44, 46] has been adapted for opioids as described further below in the Opioid Use Monitor (OUM) [47].

The Progress Assessment is another brief counselor-administered measure recently developed for MBC, including a substance use question and 5 items assessing relapse risk and 5 items assessing factors protective of relapse [48]. The aim of this measure is to personalize the content of the therapy session that immediately follows the administration of the instrument. These items are especially useful in clinical management of patients (e.g., for relapse prevention and detection), but the authors do not directly address either personalizing the dose or assessing overall syndromic severity.

The 17-item Brief Addiction Monitor (BAM) was developed about a decade ago to support MBC and outcomes assessment [49]. It is in use for MBC by the Veterans Administration and has been studied extensively. The BAM also has the benefit of software resources integrated into the EHR developed to support treatment administration at the VA and similar BAM assessment resources are also publicly available [50]. Five BAM items that demonstrated the greatest degree of change from a first to second assessment 30–60 days later were drug use, depression, craving, heavy alcohol use, and self-help involvement, but neither the five items alone nor the full BAM predicted outcomes [51].

In 2017, the National Institute on Drug Abuse Center for the Clinical Trials Network (NIDA CCTN) created a task force to investigate the BAM and several brief measures for SUD in various treatment settings: Treatment Outcomes Profile [52]; Australian Treatment Outcomes Profile [53]; Treatment Effectiveness Assessment [54, 55]; Short Drug Monitor [56]. However, no consensus was reached on the suitability of any of these specific measures in the delivery of MBC in primary or specialty care treatment of SUD and specifically OUD (RE Gore-Langton and AJ Rush, personal communication). The latter measure, the 5-Item Short Drug Monitor (SDM) was developed for a Substance Abuse and Mental Health Services Administration-funded change package encouraging MBC for AUD and other SUD as an extension of Screening, Brief Intervention, and Referral to Treatment by combining with the SDM [56]. The SDM adapted 4 PROMIS substance use items (questions 2–5), added a global measure of distress (question 1), and asked about the past 2 weeks with response choices for all items of: Never, Rarely, Sometimes, Often, or Almost Always (see Table 1). While items were generally liked by the 2017 NIDA CCTN task force, investigators in the MI-CARE trial of collaborative care for OUD and depression [57] preferred the PROMIS 7-item short form [47], discussed further below, given its demonstrated psychometric validity.

**Table 1:** 5-Item Short Drug Monitor

How often in the past two weeks …
1. Were you bothered by how your drug use impacted your health, relationships, goals or life?
2. Did you spend a lot of time using drugs?
3. Were drugs the only thing you could think about?
4. Did you disappoint yourself or others due to drug use?
5. Did you feel your drug use was out of control?

Permission to include the Short Drug Monitor [56] was obtained from the National Council for Mental Wellbeing

The US National Institute on Drug Abuse National Drug Abuse Treatment Clinical Trials Network recently produced the opioid use disorder core outcomes set (OUD-COS, version 1) in an e-Delphi consensus study that is anticipated to make NIDA CTN studies more comparable and facilitate linkage among studies, as well as align with other reporting standards [58]. The OUD-COS was finalized with five measures: Patient global impression of improvement; Non-fatal opioid overdose, Illicit/non-medical drug toxicology; Duration of treatment; and Fatal opioid poisoning.

None of the measures discussed above have yet been demonstrated to have value in either the personalized adjustment of medication dosing or as an easily used metric to determine effectiveness.

## Methods

The NIDA CCTN in 2022 convened a small workgroup to discuss measures aimed at improving the quality of treatment delivery with MBC methods for patients with OUD. The workgroup invited 10 clinicians and experts who were active in the use of MBC and/or development of treatment outcome measures relevant to SUD, some of whom had previously participated in the 2017 task force on MBC [16].

Members of the MBC workgroup were identified by the NIDA CCTN leaders and the invited workgroup hosts from NIDA CTN investigators who had expressed interest and had previous relevant experience. Background and areas of expertise of members of the workgroup are summarized below. Also see the section on Authors’ details for current affiliations.

A. John Rush, MD, is a Professor Emeritus and adjunct professor of psychiatry with clinical experience in substance use disorders treatment (esp. heroin treatment) and conducts research on innovative treatments for mood disorders including medication combinations, somatic treatments, psychotherapy, measurement-based care, disease management protocols and clinical practice guidelines. He has been an advisor to the NIDA CTN for 20 years and was host of this MBC workgroup.

Robert E. Gore-Langton, PhD, is a physiologist by training and Senior Project Leader at a clinical research organization. Recent research has focused on maternal/child health including as principal investigator/director of data coordinating centers sponsored by NICHD from 2004 to 2019. Support for the NIDA CTN since 2010 includes common data elements, clinical decision support tools for SUD and OUD, a clinical quality measure for tobacco, alcohol and drug use, and was co-host of this MBC workgroup.

Gavin Bart, MD, PhD, is an internal medicine/addiction medicine specialist, OTP director, and office-based addiction treatment provider, and conducts research with expertise in MOUD pharmacology and clinical trials for the treatment of OUD.

Katharine A Bradley, MD, MPH is a general internist who practiced primary care for over 20 years and is now a Senior Investigator who has conducted research aimed at improving care for alcohol and other substance use disorders in routine medical settings over 30 years. Her team has validated numerous practical measures to support routine medical care of patients with alcohol and other substance use and conducted pragmatic effectiveness and implementation trials of collaborative care for AUD and OUD, some using MBC.

Cynthia Campbell, PhD, MPH, is an addiction health services researcher conducting research on risks of OUD and how to improve its treatment within health care systems. Her work includes a focus on EHR-based SUD and OUD measures, and on methods using EHR data to study OUD.

James R. McKay, PhD, is a professor of psychology in psychiatry and the Director of a Center of Excellence in Substance Addiction Treatment and Education. His research has focused on developing and evaluating continuing care treatments for substance use disorders, remote delivery of SUD care via telemedicine and apps, measurement-based care, and adaptive treatments.

David W. Oslin, MD, is a professor of psychiatry and Vice Chair for Veterans Health at a Veterans Affairs Medical Center. His research portfolio includes studies aimed to improve access to behavioral health care, implementing measurement-based mental health care, and the study of pharmacogenetics of addiction and depression treatment. He is an expert in clinical trials methodology.

Andrew J. Saxon, MD, is a clinical addiction psychiatrist with more than three decades of experience including research involving pharmacotherapies and psychotherapies for alcohol, tobacco, and opioid use disorders. His work includes co-occurrence of substance use disorders and posttraumatic stress disorder, phenomenology and epidemiology of cannabis use, and treatment of substance use in primary care.

T. John Winhusen, PhD, has been a continuously funded NIDA investigator for 25 years with much of his research involving the conduct of clinical trials evaluating medication and psychosocial interventions in “real world” clinical settings. His research has focused on the treatment of stimulant use disorders and opioid use disorder.

Li-Tzy Wu, RN, ScD, MA, is a professor of psychiatry and behavioral sciences with research on measurement and nosology, validation of substance use disorder diagnoses, assessment and tool validation, collaborative care models of medication treatment for opioid use disorders, epidemiology of opioid and other drug use disorders, and research methods. She is Co-Principal Investigator of the TAPS Tool (The tobacco, alcohol, prescription medications, and other substance tool).

Landhing Moran, PhD, is a health scientist administrator at the Center for the Clinical Trials Network at NIDA, providing direction and scientific oversight for several CTN projects. She has been an investigator on clinical trials and longitudinal observational studies on substance use and OUD treatment, examining environmental influences and using ecological momentary assessment, as well as in pre-clinical research on HIV-associated neurocognitive disorders.

Betty Tai, PhD, is the founding leader of NIDA’s first and largest clinical research enterprise, the NIDA CTN. Its mission is to bridge the gap between research and practice by rapidly translating SUD science into evidence-based SUD patient care. Under her leadership the CTN has completed over a hundred trials and produced over 650 publications. The CTN has been recognized, nationally and internationally for its important contributions to the improvement of SUD patient care.

The workgroup was charged with using consensus to recommend to the NIDA Clinical Trials Network (NIDA CTN) one or more either off-the-shelf or potentially newly developed measures to address two major and related clinical tasks: (1) to reliably identify the optimal dose of medications for the treatment of OUD for each patient; (2) to estimate the effectiveness of a treatment once implemented, in a more granular fashion than the binary categories of early or sustained remission or no remission found in DSM-5 [25, 26].

The rationale for this charge was a recognition of high treatment dropout rates in patients with OUD receiving medications [59, 60]. Perhaps a more rapid, timely, personalized dosing would help retain patients by minimizing the discomfort of medication initiation. Further, a more granular assessment of outcome could enhance precision in clinical decision making so that timely revisions in the types of treatment could be made to either enhance effectiveness or retention. A universally used scaled outcome measure could assist research recognition of partially effective treatments that if sequenced or combined might lead to an effective intervention package. In addition, a universally used treatment outcome metric would help promote cross clinician, program and research study comparisons, analogous to glycated hemoglobin (HbA1c) for diabetes [61] or the Patient Health Questionnaire or other measures for depression [62].

Four virtual meetings were held on June 24, July 20, and August 5 and 31, 2022. Most but not all invited workgroup members attended all four virtual meetings. The format of each meeting consisted of a slide presentation introducing discussion topics, a summary of the preceding meeting and decisions, followed by open discussion. Summary notes of each workgroup meeting were prepared by an independent medical writer provided by the NIDA CCTN and reviewed/edited by the workgroup leadership prior to distribution to all workgroup members. Any concerns or suggestions from members were discussed when raised and/or during the next meetings. All decisions were made through open discussion, distribution of workgroup notes, and multiple rounds of review by all investigators to achieve this resulting paper. Implementation of consensus decisions was by all workgroup members (i.e., all authors) giving explicit approval for the decisions made and the wording in this paper.

To accomplish the task set by NIDA within a relatively short timeline it was necessary to begin with two main limitations on the work to be performed. First, the workgroup focused discussions on a more immediate but limited interpretation of MBC for OUD, consisting of introducing personalized dosing of U.S. Food and Drug Administration (FDA)-approved medications for OUD and determining effectiveness of these treatments on clinical outcomes. Second, workgroup discussions of measures for MBC were based on domains and widely accepted principles that were considered essential in the short or intermediate term and did not include other additional aspects that may be important in the long-term outcome of patients, such as relapse prevention or quality of life (QoL).

## Discussion

### Sharpen the focus

Our discussions revealed the need to clarify and focus our efforts to achieve consensus. The following summarizes the questions raised, decisions made and the supporting rationale.

#### Which aspects of measurement-based care (MBC)?

Many people view MBC as entailing the regular use of simple clinical ratings and ordinary laboratory tests to accomplish a variety of clinical tasks including selecting a treatment, personalizing treatment dosing, minimizing side effects, and making timely changes in treatment in the face of inadequate response [63]. Typically, MBC methods are applied at the clinician-patient interface, but other stakeholders (e.g., administrators) may also use the same measures to manage program resource allocation. Researchers and regulators also want measures to assess the safety and efficacy of treatments and quality of care. For purposes of brief MBC in primary care or specialty care, in this workgroup we focused on measures to enhance dosage optimization and timely revision in treatments when outcome is suboptimal.

We found that we needed to clarify the conceptual distinctions between MBC approaches typically defined by and designed for various clinical tasks as noted above: treatment sequences—sometimes called algorithms—and disease management protocols.

Treatment algorithms define the principles, rationale, and evidence for which treatment to initiate and which to follow depending on therapeutic and adverse effects achieved with the prior treatment steps [64]. The algorithm may recommend one or a range of reasonable treatment options at each step. The combination of measurement of outcomes at each step and a systematic organization of the steps is associated with better outcomes and less expensive care in depression [64, 65] and other conditions such as panic disorder, generalized anxiety disorder, bipolar disorder, etc. [11, 14].

Disease management protocols are population-based efforts that have a broad set of components designed to improve chronic disease outcomes. They often recommend procedures to screen for, establish the history, and define a differential diagnosis. These protocols also typically recommend treatment options, ways to personalize treatment titration or delivery, recommend treatment algorithms, specify multi-dimensional approaches to enhancing symptom reduction, relapse prevention, functional restoration, and minimization of side-effect burden, as well as addressing issues of adherence and lifestyle changes. Disease management protocols often involve multiple treatment team members.

**Fig. 1:**
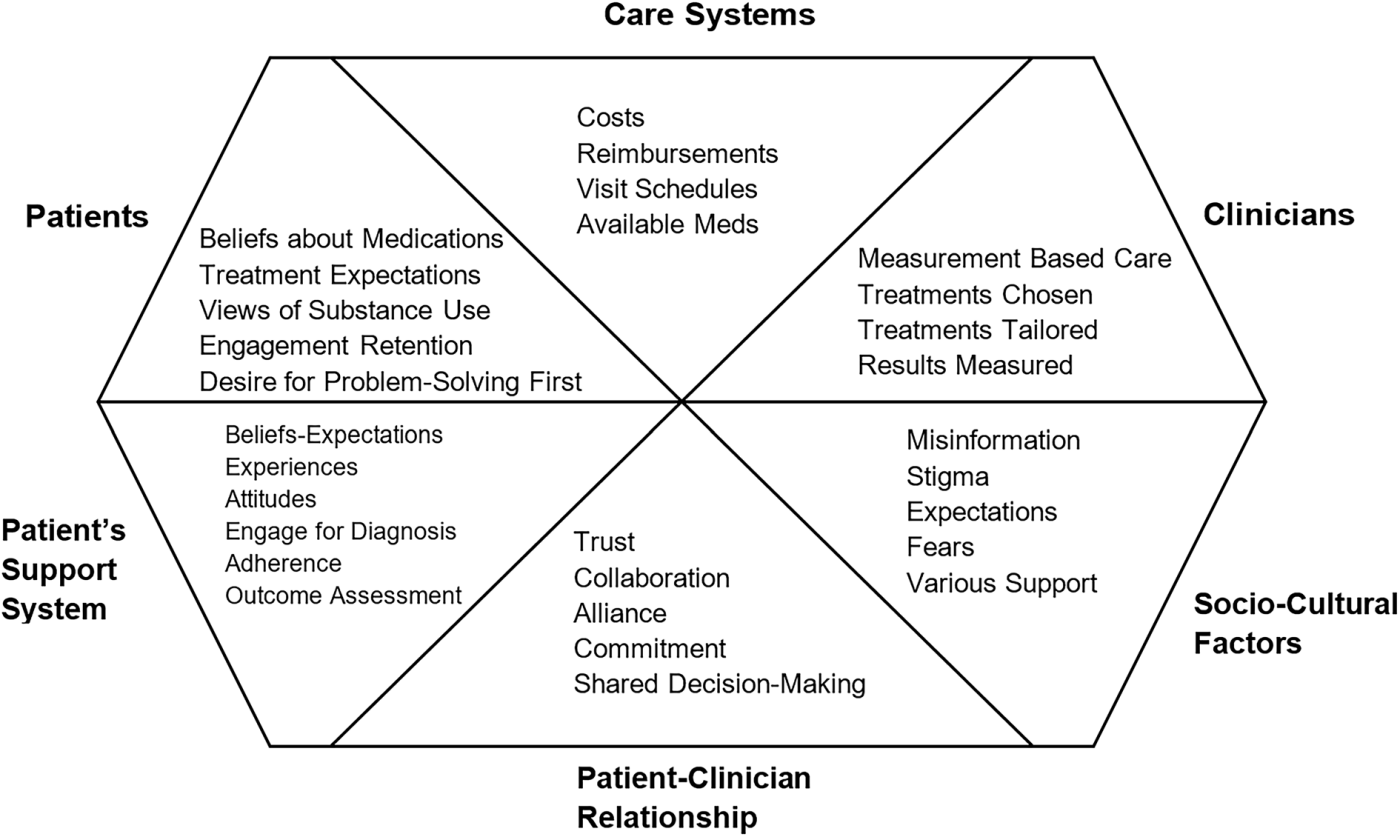
Factors affecting treatment outcomes. Adapted with permission from the *American Journal of Psychiatry*, Volume 175, Issue 12, “Improving Depression Outcome by Patient-Centered Medical Management,” A. John Rush and Michael E. Thase, p. 1188. (Copyright © 2018) [66]. American Psychiatric Association. All Rights Reserved

Our discussions led us to provide a context for MBC as shown in Fig. 1. However, MBC is only one ingredient within a complex array of factors that affect outcomes.

**Fig. 2:**
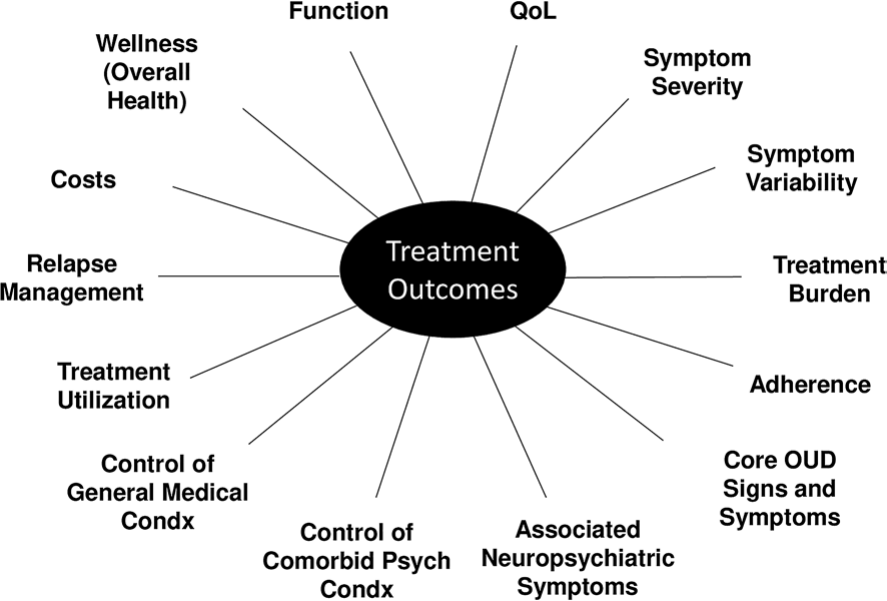
Core OUD signs and symptoms. Permission to adapt this figure [67] was obtained under the terms of the Creative Commons CC BY license

In addition to the host of parameters that affect treatment outcomes, Fig. 2 shows the range of important treatment outcomes of interest. Consensus formed around the idea of measuring those outcomes that were specific to OUD and that were clinically most relevant to assessing the effectiveness of the treatment and its rapid and comfortable dosing obtained at the patient-clinician interface in primary care or specialty care. We reached this consensus for two reasons. First, the effectiveness of MBC has been most robustly demonstrated when it has been used to adjust medication doses and to assess symptomatic treatment outcomes, for example in mental health arenas such as depression [11–13]. Second, other measures are likely needed to accomplish different clinical tasks or to assess whether other outcomes were achieved, such as predicting relapse.

#### Phases of treatment

There was a consensus to recognize that roughly speaking the phases of OUD treatment approximate those outlined by Rush and Thase [66] for the patient-centered delivery of medications for depression (see Table 2). The measure being considered for medication dose personalization would typically be used early in treatment, whereas the measure to assess overall treatment outcome would be used somewhat later.

**Table 2:** Patient-Centered Treatment Phases

Engagement & Retention	• Establish collaboration • Align expectations • Agree on goals & metrics
Adherence	• Develop realistic expectations
Symptom control	• Shared decision making • Tailor treatment (MBC)
Functional restoration	• Redress relationships, work
Relapse amelioration	• Resilience training; Stress management • Prodromal symptoms management

Adapted with permission from the *American Journal of Psychiatry*, Volume 175, Issue 12, “Improving Depression Outcome by Patient-Centered Medical Management,” A. John Rush and Michael E. Thase, p. 1189 (Copyright © 2018) [66]. American Psychiatric Association. All Rights Reserved

#### Which disorders: OUD or SUD?

While we initially aimed at SUD, we found that an initial focus on medication treatment for OUD would likely be more productive because the types of treatment being dose adjusted will vary across different SUDs and because of greater availability of FDA-approved medications for OUD.

#### Core symptoms or associated symptoms?

We discussed whether to focus on DSM-5 *core symptoms of OUD* or to also include symptoms of commonly associated conditions, such as pain, sleep, anxiety, depression, etc. The focus on core OUD symptoms was chosen because the other conditions are likely more useful in tailoring other aspects of OUD care (e.g., counseling) and are often already measured in well-validated brief measures in primary care and specialty settings.

By focusing on core DSM-5 symptoms of OUD, we avoid the inadvertent measurement of associated symptoms that occur in the context of concurrent SUDs. We recognize that polysubstance use that involves misuse of other substances is common in those with OUD, which can adversely affect patient outcomes and undermine the sustained impact of OUD medications, even if opioid use is initially reduced by properly dosed medications for OUD. Given that broad assessment of these other factors is not compatible with a brief MBC measure, we urge providers to inquire about factors that may be undermining outcomes in situations where a medication dose that was working well is suddenly not preventing opioid use.

In support of using DSM-5 criteria for OUD to assess treatment outcomes, recovery subgroups from buprenorphine treatment at long-term follow-up assessment have been aligned with DSM-5 OUD severity criteria (past 3 months) and OUD outcomes [68]. Also, a systematic review and meta-analysis suggests the use of a measure of craving, one of the diagnostic criteria of DSM-5, to estimate risk of drug use or relapse for assessment and treatment [69]. Further investigation of DSM-5 core symptoms for assessment and treatment is still needed.

#### Which interventions?

We chose to focus initially on medication treatment because it can be provided in primary care and specialty care settings. Further, because pharmacological interventions need to be initiated and adjusted in ways that are distinct from psychosocial interventions, rather than attempting to develop scales that would be suitable for both types of interventions, a focus on psychopharmacological intervention seemed a feasible first step. Nevertheless, a measure of core symptoms, if agreed to, should be as useful in assessing the effects of psychosocial, medication and even device-based interventions on OUD.

#### Which clinical settings?

The intention was to identify measures that would help clinicians rapidly and accurately personalize the doses of OUD medications when initiating medications *and* assess treatment outcome suitable for use in both primary and specialty care settings by clinicians, patients, and families. In OTP settings where treatment adjustments are protocolized MBC may allow for better tailoring of dose adjustments to larger increments in dose at different frequencies depending on the individual patient.

We agreed that if more extensive/longer measures of outcomes were needed, say in specialized care settings, they could be added to the core outcome measure. A single measure facilitates patient management and communication among clinicians and across settings (e.g., programs that have expert primary care clinicians initiate medications and then hand them off to primary care providers when the patient is stable, or for “hub and spoke” OUD treatment systems [70]), as well as the compilation of programmatic outcomes by administrators. Such a universally agreed to measure could be part of the core outcome package for research studies of OUD treatments, facilitating the transfer of knowledge between researchers and clinicians.

#### Who would be using these measures?

We chose to focus primarily on *measures for routine clinical care, as opposed to the research users* because researchers could and often need to include longer or additional measures not easily administered in routine care. Patient self-report measures of substance use symptoms and brief screens for SUD have demonstrated validity in primary care even when results are documented in the EHR [23, 29, 30, 71]. Our concern was optimizing doses and obtaining accurate, practical, and timely assessment of treatment outcome, rather than achieving a complete understanding of all the obstacles to and opportunities for patient improvement.

#### Two measures or one?

We initially hoped to have a single measure that could be used for personalization of medication induction and dosing and as a reasonably precise measurement of overall OUD treatment effectiveness. During our discussions we realized that measures to accomplish these two clinical tasks (personalized medication dosing; assessment of treatment effectiveness) might both overlap in terms of some signs and symptoms and not overlap in terms of others.

Dose adjustments (which occur within 1–3 weeks of initiating medication) precede the assessment of overall treatment effectiveness. Treatment effectiveness may not be fully known for weeks to months. That is, these two tasks may be completed at different times. Secondly, parameters that best inform dose adjustment, may or may not be elements that are most useful in assessing treatment effectiveness. That is, both when to measure and what to measure to bring precision to each clinical task are likely to be distinct. This conclusion initially supported the notion of developing two metrics, one for each task.

#### What are the desirable features of a treatment outcome measure?

There was a consensus that having both self-report (for use in the clinic) and clinician-completed versions (for research and regulatory approval) of any measure would be very useful and that crosswalks should be created between such instruments. Self-reports can promote patient and family participation in treatment and can be used outside the treatment site (e.g., by smart phone). Clinician ratings may bring greater between-patient reliability. In addition, clinician ratings can be compared with self-reports, especially if the items are identical. We recognize that the accuracy of both self-reports and clinician ratings (both of which typically rely on patient reporting) is highly influenced by patient recollection and forthrightness.

There was a consensus that both dose adjustment and outcome measures must be brief—preferably 5–6 and no more than 9 items to facilitate ease of use and portability to smart phones. A global (i.e., brief, complete) rating such as the widely used pain scale or the Clinical Global Impression of Severity measure [72] may suffice in many instances.

#### Is measurement-based care for OUD more effective than treatment as usual?

Logically, more precise measurement to personalize medication doses or to establish treatment effectiveness, might bring greater precision to clinical decision-making in the acute management of patients with OUD and greater consistency in their care amongst practices and clinicians. This in turn could improve outcomes (e.g., greater engagement and retention in treatment, lower amounts, and less frequent opioid use, better daily function and QoL, less frequent severe or prolonged relapses, etc.). However, there is yet no randomized controlled trial evidence that MBC approaches when applied to OUD medication management are more effective than treatment as usual. There is evidence from a meta-analysis that higher buprenorphine dose is associated with improved retention in buprenorphine maintenance treatment [73]. There was consensus that if these measures could be agreed to, the impact of MBC in OUD patients must be assessed to determine whether there is any advantage to this approach in patients with OUD given opioid agonists or other treatments.

### Domains for dose adjustment and assessment of effectiveness

This section details our discussion about the elements/parameters or domains that seem to be essential to accomplishing each task: (1) OUD medication *dose adjustment* for each patient (personalized dosing) and (2) assessment of acute *treatment effectiveness* on the core signs and symptoms of OUD.

#### Medication dose adjustment

MBC when applied to dose personalization requires that both side-effect burden and the therapeutic effect be assessed to identify the optimal cost benefit balance for each patient. Personalized dosing may be required because some medications need to be given at higher doses to some patients and at lower doses to others due to pharmacokinetic and pharmacodynamic variability across patients and differing tolerance because of previous drug usage.

The following five parameters were considered sufficient measures for medication dose adjustment: withdrawal symptoms, opioid use, magnitude (severity and duration) of the subjective effects when opioids are used, craving, and side effects. The consensus was for a brief rating of the 4 OUD-specific parameters (either by a few separate items or a global overall assessment) to adjust dosing, and a global assessment or verbal question for side effects. In addition, adherence to the prescribed medication must be checked at each patient-clinician interaction, potentially informing switching to a different medication or formulation (e.g., methadone, long-acting buprenorphine, etc.). It is expected that the psychosocial consequences such as failed role obligations, opioid use despite social problems, and the discontinuation of important activities may NOT change as substantially/meaningfully during this initiation period of 1–3 weeks [74, 75] as during the ensuing weeks to months.

#### Assessment of treatment effectiveness

Given the prior work done by Marsden and colleagues [16] (“Marsden measure”) and Bradley and colleagues [47], and the usual approaches to assessing treatment outcomes in other psychiatric and general medical conditions, we focused our initial discussions on the criterion signs and symptoms that define OUD based on DSM-5-TR [26]. Table 3 synopsizes the Marsden measure [16] which recommended 5 conceptually separate domains that were adapted from the 11 DSM-5-TR criteria for OUD [26].

**Table 3:** Domains adapted from DSM-5 Substance Use Disorder Criteria

Domain	Metric
A. Use	• Frequency, types and amount used
B. Physiological	• Need for higher doses • Withdrawal
C. Cognitive/Behavioral	• Use more often, or longer than intended • ailed quit attempts • Time spent obtaining, using, recovering • Urges (bothersome), craving
D. Health Risks	• Use in hazardous situation • Use with medical damage knowledge
E. Negative Social Consequences	• Failed role obligations • Use despite social problems • Discontinue important activities

Permission to adapt this table [16] was obtained under license from John Wiley and Sons

The first four domains (A–D) of the Marsden measure [16] are specific to OUD because opioids are specified in the individual question items, although should be generalizable to similar addicting drugs: the frequency type and amount of use of an opioid (A); the physiological manifestations of tolerance or dose reduction of an opioid (B); the subjective experiences in terms of desire, control, and urges/craving for these kinds of substances (C); and the use of opioids in dangerous circumstances (D). We recognize that domain D, while specific to opioids, will be variably applicable to patients depending on their type of use and social circumstance.

Domain E of the Marsden measure [16] about negative social consequences, broadly includes daily function and QoL and is not specific to OUD. Negative social consequences are applicable to any SUD and indeed, most other neuropsychiatric and many general medical conditions. Further, negative social consequences can be caused by other concomitant SUDs and general medical and psychiatric conditions (e.g., chronic depression, pain, sleep wake disorders, etc.). An additional reason for separating the measurement of core OUD symptoms (Domains A–C) and function/QoL (Domain E) is that the latter is very likely to improve after the signs and the symptoms (A–C) of OUD are brought under some degree of control for a meaningful period, as suggested by some studies [74, 75].

Separating the assessment of function and QoL (negative social consequences), as in the Marsden measure, would allow for the use of any of a host of brief, psychometrically sound scales that are widely accepted assessments of function and quality of life, though less often applied to SUDs or OUD to date. The workgroup consensus was that measurement of function and quality of life (which would include negative social consequences) separately from the core OUD signs and symptoms per se would have some major advantages.

Bradley and colleagues’ preliminary work for the More Individualized Care: Assessment and Recovery Through Engagement (MI-CARE) trial [76] illustrates the application of these principles in their adaptation of the PROMIS Item Bank v1.0—Short form 7a substance use measure [44, 47] and a psychometrically validated substance use symptom checklist [30]. The OUM was adapted from the substance use PROMIS measure by substituting “opioids” for “drugs.” The baseline timeframe was 3 months but the time frame for follow-up was the past 2 weeks to support measurement-based care [47]. Seven items of the OUM are each rated on a 0–4-point scale using the PROMIS response options (“never” to “almost always”; see Table 4) yielding a total score of 0–28. This scale assesses a range of outcomes. Six of the seven items assess the signs and symptoms of OUD, while only one assesses the effect of the OUD on interpersonal relations. Some items (e.g., “I have an opioid problem”) may need to be revised or potentially deleted if not responsive to change. This measure is expected to reflect the overall severity of the OUD [44] and initial results in a sample of 49 primary care patients made it appear feasible with variability in responses [47].

**Table 4:** PROMIS-based Opioid Use Monitor (OUM)

1. I felt that my opioid use was out of control	SCALE
2. My desire to use opioids seemed overpowering	0 = Never
3. Opioids were the only thing I could think about	1 = Rarely
4. My opioid use caused problems with people close to me	2 = Sometimes
5. I have an opioid problem	3 = Often
6. I craved opioids	4 = Almost always
7. I spent a lot of time using opioids	

*Thinking about the past 2 weeks, please select the one best answer for each question.* This table was adapted from PROMIS [44, 47] under Public Domain

### Summary and next steps

#### Medication dose personalization

A consensus was developed indicating that a brief rating—either a global rating or a few separate questions that assess the severity of withdrawal symptoms, craving, amounts of opioids used, and subjective response to use of opioids during treatment and a single item global rating of side effects are sufficient to adjust the dose of agonists to each patient.

Whether the use of such a 4-element tool or a global rating to personalize dose adjustment of agonists in the treatment of OUD produces better retention, good patient or clinician satisfaction, and/or different “final” doses deserves study as compared to treatment as usual without any measures.

While no specific brief measure incorporating all four of these elements was identified or recommended, as an example the PROMIS-based 7-item OUM used in the MI-CARE trial described above to assess symptom severity and support MBC includes questions pertaining to each of the recommended dose-personalization elements except for withdrawal [47]. Further, the OUM includes 3 craving questions, which are used as a separate craving score (0–12) in the registry of the trial. The usefulness of the OUM craving questions suggests that an off-the-shelf craving tool could also be considered for the dose adjustment portion of MBC but would require research to determine if it is sufficient. The possibility of adapting an existing tool or developing a new tool with the recommended elements for medication dose personalization should be considered.

#### Assessment of therapeutic effects

There was consensus that core signs and symptoms of OUD based on some of the 5 DSM-5 domains in the Marsden measure [16], should be the basis for assessing treatment outcome. A select list (e.g., 5–7 items) of these core symptoms and signs rated over the prior 1–2 weeks and used after a clinically reasonable period on a treatment (e.g., 1–2 months) would determine the effect of the treatment on the condition (OUD), such that a clinical decision could be informed by the outcomes obtained (e.g., whether to change the treatment, augment it, etc.).

Domains A and B (see Table 3) of the five DSM-5 domains from the Marsden measure [16] also pertain to dose adjustment. Further, the OUM, discussed above for dosing adjustment, is also appropriate for assessing treatment outcomes, so it is possible that adaptations of a single measure could serve both functions, although maybe not optimally, or that there be two separate optimal measures.

#### Other considerations and next steps

There was consensus that measurement of function and quality of life (including negative social consequences) should proceed independently with brief validated measures that are widely used in mental health and general medical conditions. The performance of these measures in patients with OUD deserves study.

There was agreement that if more than one outcome measure was recommended or developed, these measures should be assessed and when possible, crosswalks established between them in terms of total severity. Each measure should be assessed in different populations and age groups, but we should avoid tailoring different instruments to different sociodemographic or clinical subgroups. Nevertheless, care should be taken in the measure development and validation processes to consider and assess potential disparities in measure performance in different patient subgroups such as race, ethnicity, age, chronicity, culture and lived experience.

Next steps could be to select and adapt or develop de novo brief measures for OUD treatment medication dose personalization and treatment effectiveness assessment along the lines previously discussed that include the consensus recommended domains and the signs and symptoms of OUD. Whether the use of these measures in clinical care of patients with OUD produces better outcomes (e.g., retention, less drug use, better symptom control, better QoL and daily function or better prognosis) as compared to treatment as usual without measurements deserves investigation. None of the existing measures could be fully recommended to accomplish these aims for OUD because of lack of clinical evidence, absence of certain essential (i.e., recommended) domains, and/or excessive length and complexity. However, the PROMIS-based measure currently in use for OUD (i.e., the OUM) [47] could be considered for adaptation. Alternatively, a brief measure for dose personalization could be developed de novo*,* or the evaluation of the clinical impact by a single-question global scale that encompasses the 4 suggested parameters could determine whether MBC has clinical utility in arriving at the personalized dose in an expeditious fashion*.* Once the measures to personalize dosing and assess treatment effectiveness are developed or agreed to by experts, patient acceptability and psychometric studies are also needed.

## Disclaimer

The views and opinions expressed in this manuscript are those of the authors only and do not necessarily represent the views, official policy, or position of the US Department of Health and Human Services or any of its affiliated institutes or agencies.

## Data Availability

Data sharing is not applicable to this article as no datasets were generated or analyzed during the current study.

## Abbreviations

AHCPR: Agency for Health Care Policy and Research
AMNet: The Addiction Medicine Practice-Based Research Network
AUD: Alcohol use disorder
BAM: Brief addiction monitor
CDE: Common data element
DSM-5: The Diagnostic and Statistical Manual of Mental Disorders, fifth edition
DSM-5-TR: DSM-5 text revision
EHR: Electronic health record
FDA: U.S. Food and Drug Administration
HbA1C: Glycated hemoglobin
KP: Kaiser Permanente
MBC: Measurement-based care
MI-CARE: More Individualized Care: Assessment and Recovery Through Engagement trial
MOUD: Medications for opioid use disorder
NICHD: *Eunice Kennedy Shriver* National Institute of Child Health and Human Development
NIDA CTN: NIDA’s National Drug Abuse Treatment Clinical Trials Network
NIDA CCTN: NIDA’s Center for the Clinical Trials Network
OPT: Opioid treatment program
OUD: Opioid use disorder
OUM: Opioid use monitor
PROMIS: Patient-Reported Outcomes Measurement Information System
QoL: Quality of life
SAMHSA: Substance Abuse and Mental Health Services Administration
SDM: Short drug monitor
SPARC: Sustained Patient-Centered Alcohol-Related Care trial
SUD: Substance use disorder
TLFB: Timeline follow-back
TOP: Treatment outcomes profiles
